# Red Emission Carbon Dots Prepared by 1,4-Diaminonaphthalene for Light-Emitting Diode Application and Metal Ion Detection

**DOI:** 10.3390/ma14164716

**Published:** 2021-08-20

**Authors:** Yulong An, Xu Lin, Zewen Guo, Qitao Yin, Yan Li, Yunwu Zheng, Zhengjun Shi, Wuxian Zhang, Can Liu

**Affiliations:** 1 Key Laboratory for Forest Resources Conservation and Utilization in the Southwest Mountains of China, College of Materials Science and Engineering, Southwest Forestry University, Kunming 650224, China; ayl96yu@163.com (Y.A.); w2844207536@163.com (Z.G.); xjyqt123456@163.com (Q.Y.); ly97111659@163.com (Y.L.); 2 Key Laboratory of State Forestry Administration for Highly-Efficient Utilization of Forestry Biomass Resources in Southwest China, Southwest Forestry University, Kunming 650224, China; zyw85114@163.com (Y.Z.); shizhengjun1979@swfu.edu.cn (Z.S.); 13700687552@swfu.edu.cn (W.Z.)

**Keywords:** carbon dots, solvothermal method, tunable emission, 1,4-diaminonaphthalene, solvents effect

## Abstract

Carbon dots (CDs), as the most important type of carbon materials, have been widely used in many fields because of their unique fluorescence characteristics and excellent properties of biocompatibility. In previous studies, the fluorescence of CDs was mainly concentrated in the blue and green, whereas the red fluorescence was relatively less. Herein, we prepared efficient red-emitting CDs from 1,4-diaminonaphthalene using solvothermal methods. We discussed the effects of different solvothermal solvents on CDs. The results show that CDs prepared with octane and acetone as reaction media have the best fluorescence properties. The CDs dispersed in different organic solvents exhibited tunable emission across a wide spectrum from 427 nm to 679 nm. We further demonstrated the application of red light-emitting diode (LED) optoelectronics and fluorescence detection of Fe^3+^ in aqueous solution.

## 1. Introduction

Since their first discovery in 2004, carbon dots (CDs), as a new breakthrough in the field of carbon nanomaterials [1], have attracted extensive attention in the past decade. Compared with traditional semiconductor quantum dots, CDs have good biocompatibility, high photoluminescence quantum yield, low production cost and an environment-friendly synthesis method [2,3,4,5]. These characteristics provide CDs with great application potential in the fields of sensors, display lighting equipment and biology [6,7,8,9].

In previous studies, the emission wavelengths of fluorescent CDs are mainly concentrated in the blue, green, and yellow-orange emission regions [10,11,12], whereas the red fluorescence is relatively less [13,14,15]. In addition, the development of photoluminescence in the whole visible region is very important for the practical application of CDs [16,17,18,19,20,21]; therefore, attempts to produce CDs with tunable emission have rarely been reported [22,23]. When the carbon source and reaction solvent are properly adjusted, the fluorescent CDs can be obtained by a solvothermal method covering all visible light emission [24,25,26]. Ding et al. [8] prepared a batch of CDs by hydrothermal synthesis with *p*-phenylenediamine and urea as raw materials. They separated CDs by silica gel column chromatography and obtained eight groups of CDs, with luminescence ranging from blue to red (440 nm–625 nm). Using *p*-phenylenediamine as the carbon source, Zhang et al. [27] also prepared relatively efficient red-emitting CDs using different solvothermal methods. The CDs displayed different color fluorescence in different solvents. In addition, tunable solid-state fluorescence can be achieved in the range of 545 nm to 595 nm by adjusting the proportion of matrix in the polymer. However, there are still many problems in the synthesis of CDs, such as complex synthesis methods, a limited source of raw materials, and other factors. Therefore, developing simple CDs synthesis methods is still a great challenge.

In the present work, we prepared efficient red-emitting CDs from 1,4-diamine naphthalene using solvothermal methods. Compared with the reported *p*-phenylenediamine, 1,4-naphthalene diamine has a larger conjugated structure, which provides the possibility of preparing efficient fluorescent CDs. We also discussed the effect of different reaction solvents on the CDs. The results show that CDs prepared with octane and acetone as reaction media have the best fluorescence properties. Furthermore, we also report the tunable luminescence of CDs in different organic solvents. In various solvents, the maximum fluorescence wavelength of the CDs can be adjusted from 427 nm to 679 nm. In addition, using the fluorescent properties of the CDs, we further demonstrated the application of fluorescence detection of Fe^3+^ in aqueous solution. Finally, red fluorescent light-emitting diodes (LEDs) were prepared by coating CDs/epoxy resin composites on light-emitting diode chips with a 365 nm wavelength.

## 2. Materials and Methods

### 2.1. Materials

Column chromatography was performed using 200–300 mesh silica gel. 1,4-diaminonaphthalene (98.0%), octane (99.0%), toluene (99.0%), hexanol (99.0%), methanol (99.0%), tetrahydrofuran (THF, 99.0%), ethanol (99.7%), and epoxy resin (epoxy value: 0.46–0.5 mol/100 g, viscosity: 1100–1600 mPa·s) were purchased by Shanghai Titan Scientific Co., Ltd. (Shanghai, China). Dichloromethane (99.0%), acetone (99.5%), and chloroform (99.0%) were provided from Shandian Chemical (Yunnan, China). *N*,*N*-dimethyl formamide (DMF, 99.5%) was provided by GHTECH (Guangdong, China). All other commercially available reagents and solvents were of reagent grade and were used without further purification.

### 2.2. Methods

Transmission electron microscopy (TEM) images observation was performed with FEI Tecani G2 F20 TEM (FEI, Hillsboro, OR, USA) operating at 200 kV accelerating voltage. UV-vis spectra in the liquid state were recorded with an ultraviolet-visible spectrophotometer (Shimadzu UV-2600, Shimadzu, Tokyo, Japan). Fluorescence spectra of the liquid materials were measured on Shimadzu fluorescence spectrophotometer RF-6000 (Shimadzu, Tokyo, Japan). Nanosecond fluorescence lifetime was measured by the time-correlated single photon counting (TCSPC) system (HORIBA Scientific iHR 320, Paris, France). X-ray photoelectron spectroscopy (XPS) was performed on Thermo Fisher Scientific K-Alpha (Thermo Fisher Scientific, Waltham, MA, USA). Raman spectra were obtained using HORIBA Scientific LabRAM HR Evolution (HORIBA Scientific, Paris, France). 

Absolute quantum yield (QY) measurements were performed in a FLS1000 spectrometer (Techcomp, Livingston, Edinburgh, UK) equipped with a calibrated integrating sphere. We conducted the light test from a FLS1000 spectrometer to the sphere. The QY was determined by the ratio between photons emitted and absorbed by CDs. The aqueous solution of CDs was placed in a cuvette to measure its QY, while the solvent water was used as a blank sample for the reference measurement.

### 2.3. Synthesis of CDs

1,4-naphthalene diamine (0.10 g) was dissolved in 10 mL reaction solvents (octane, toluene, ethanol, acetone, and DMF), and then the solution was transferred to an autoclave lined with polytetrafluoroethylene. The autoclave was heated at 180 °C in a muffle furnace, kept for an additional 12 h, and naturally cooled to room temperature to obtain crimson suspensions. The precipitates were removed and vacuum filtered to obtain a crimson solution. After the solvent had evaporated, purification by silica gel column chromatography (eluent: dichloromethane: ethanol = 9:1) presented R-CDs as a dark brown solid (26 mg, 26%, for CDs-1; 18 mg, 18%, for CDs-2; 21 mg, 21%, for CDs-3; 21 mg, 21%, for CDs-4; 19 mg, 19%, for CDs-5).

### 2.4. Preparation of CDs-LEDs

In detail, 1.0 mg CDs were mixed with 4.0 mL epoxy resin thoroughly, and then the mixture was drop-casted on the 365 nm emissive UV-LED chip (the voltage of 3.4 V). The mixture was horizontal and smooth on the surface of the chip without bubble doping. The chip was placed in an oven at 80 °C for 3 h. Finally, in order to make LED devices, the UV chip was fixed on the LED base.

### 2.5. Fluorescence Detection of Fe^3+^

In a typical assay, a series of FeCl_3_ solutions with concentrations of 0, 20, 40, 100, 200, 300, 400, 500, 600, 700, 800, 900, and 1000 µM were prepared in Tris-HCl solution (pH 7.0) and 10 mg of CDs was dissolved in 10 mL of Tris-HCl solution. Then, 45 μL of a certain concentration of Fe^3+^ was added into 5 µL CDs solution and incubated at room temperature for 10 min to record the fluorescence emission spectrum at 610 nm. To evaluate the selectivity towards Fe^3+^, different metal ions, such as Cu^2+^, Al^3+^, Mg^2+^, Mn^2+^ and Li^+^, with the solution of 100 μM, were added into the CDs solution in a same method. All measurements were performed in triplicate. All the solutions of metal ion were made from their chloride.

## 3. Results

### 3.1. Optical Properties

**Figure 1:**
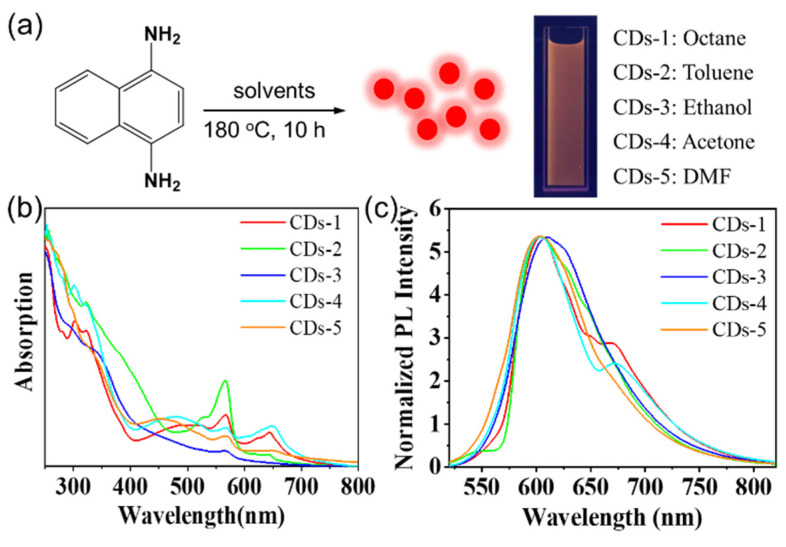
(**a**) Preparation of red-emitting CDs from 1,4-diaminonaphthalene. (**b**) UV/Vis absorption and (**c**) PL emission spectra of CDs (0.1 mg/mL) in ethanol.

We prepared red-emitting CDs by the solvothermal method, in which 1,4-diaminonaphthalene was reacted in octane (CDs-1), toluene (CDs-2), ethanol (CDs-3), acetone (CDs-4), and DMF (CDs-5) (Figure 1a). The resulting CDs demonstrate excellent solubility in common solvents, such as ethanol and chloroform, but they are insoluble in water. The CDs can be dispersed in ethanol to form a homogeneous solution, which can be seen glowing bright red in ultraviolet light. We measured the UV–visible (UV/Vis) spectra of CDs in ethanol (Figure 1b). The high-energy region and significant absorption peak are observed at around 310 nm for these CDs, which are attributable to the n–π* transition of the oxygenous functional groups. In the lower-energy region, two absorption peaks are observed at 567 nm and 647 nm, which were usually assigned to the surface-defected state transition. The CDs-1 (octane) and CDs-4 (acetone) exhibited the best absorption in the 647 nm low-energy region.

**Figure 2:**
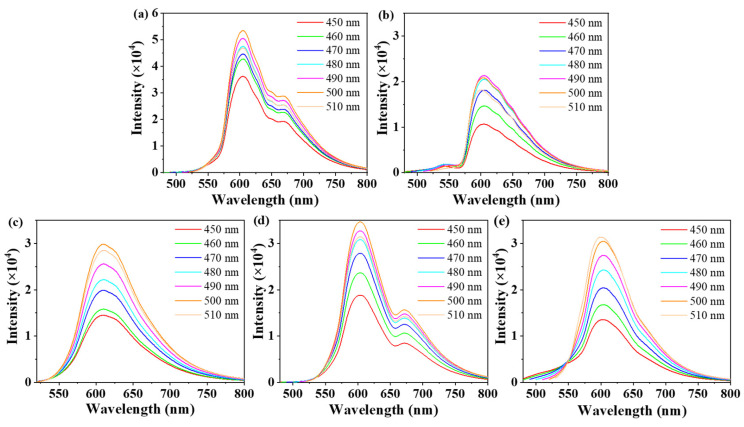
(**a**–**e**) PL emission spectra of CDs-1 to CDs-5 (0.1 mg/mL) in ethanol.

**Figure 3:**
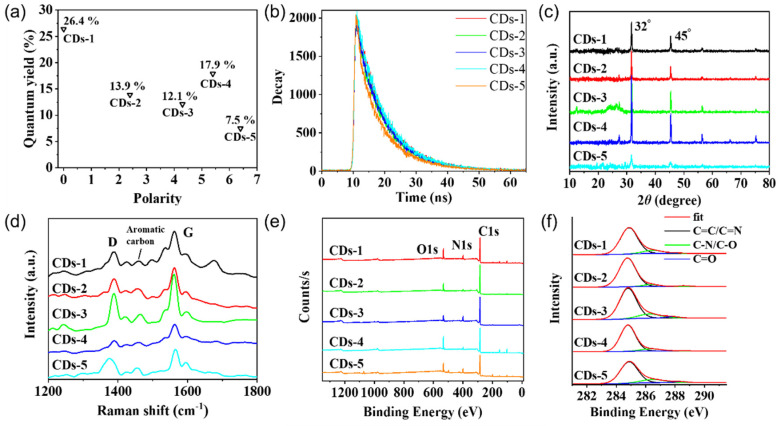
(**a**) Scatter diagram of the carbon dot quantum yields vs. the polarity of the reaction solvent. (**b**) Photoluminescent decays of CDs-1 to CDs-5 (0.1 mg/mL) in ethanol. XRD patterns. (**c**) Raman spectra. (**d**) XPS survey spectra. (**e**) High-resolution C1s spectra (**f**) of CDs-1 to CDs-5.

We measured their photoluminescent (PL) spectra in an ethanol solution. As shown in Figure 1c, the maximum emission peak of CDs-1 to CDs-5 in ethanol solution varies over the range 605–610 nm at the single exciting wavelength *λ*_ex_ = 500 nm. The PL spectra CDs excited with *λ*_ex_ = 450–510 nm are shown in Figure 2a–e, and they show the classical excitation-independent emission characteristics [27,28]. The maximum emission peak did not change significantly in these CDs, which indicate that the different solvents have no effect on the maximum emission peak. CDs-1 and CDs-4 showed relatively high QYs, which were 26.4% and 17.9%, respectively. In addition, the QYs of CDs-2 and CDs-3 were 13.9% and 12.1%, respectively. CDs-5 showed the lowest QY (7.5%). Figure 3a shows the inverse relationship between the QYs and polarity of reaction solvents. Except for acetone, their QYs basically decrease with the increase in the polarity of the reaction solvent. Octane, as a non-polar solution, shows a higher fluorescence quantum yield promotion effect on the performance of CDs than the polar solution. We also measured the attenuation kinetics of the fluorescence of CDs-1 to CDs-5, as shown in Figure 3b; all the CDs showed a single exponential decay [29], and the fluorescent lifetimes of CDs-1 to CDs-5 were 9.03, 8.85, 8.85, 8.86, and 8.34 ns, respectively.

### 3.2. Surface Characterizations and Morphology

The powder X-ray diffraction (XRD) data show that these CPDs exhibit their peak at 32°, which corresponds to the (100) crystal plane of graphite (Figure 3c). The Raman spectra show an ordered G band at 1554 cm^−1^ and a disordered D band at 1382 cm^−1^. The ID/IG ratios of CDs-1 to CDs-5 are 0.43, 0.68, 0.46, 0.43, and 0.76, respectively, suggesting the high degree of crystallinity of the five CDs (Figure 3d) [27]. We studied the functional groups of CDs-1 to CDs-5 with X-ray photoelectron spectroscopy (XPS) to determine the group distribution characteristics of these CDs samples. As shown in Figure 3e, the results show that the elemental composition of the different CD samples are similar, and the nanomaterials contain three main elements: C1s (284.7 eV), N1s (398.8 eV) and O1s (532.4 eV) [28,30,31]. This result also shows that there are significant differences in the proportions of the elements in the different samples. CDs-1 and CDs-4 have the highest XPS strength at O1s (Table 1), which means that there are a lot of oxygen-containing functional groups (such as C–O–C, C=O, and –OH) on the surface of CDs [32]. On the other hand, the resolved C1s spectra show peaks at 284.8, 286.4, and 288.2 eV in the high-resolution XPS spectra (Figure 3f), indicating the presence of C=C/C–C, C–N/C–O, and C=O, respectively [27,33]. This XPS analysis, combined with the peak-area ratios of the chemical bonds in the different CD samples, confirms that CDs-1 and CDs-4 contain the lowest number of C=O functional groups (Table 1). According to previous reports, electron-withdrawing groups, such as C=O groups, weaken the light-emitting, whereas electron-donating groups, such as C–O–C groups, promote the light-emitting [32]. Thus, the high QYs of CDs-1 and CDs-4 can be attributed to their excellent surface state, with more C–O–C groups and fewer C=O groups. Certainly, the conjugation effect and surface state jointly determine the photoluminescence of CDs. However, through XPS analysis, we can recognize that all five CDs have a high content of conjugated structure (nearly 90%), which may be related to the preparation of naphthalene. In this situation, the surface state of CDs is particularly important for photoluminescence of CDs. Therefore, CDs-2 with the highest content of conjugated structure cannot show the highest QY.

**Figure 4:**
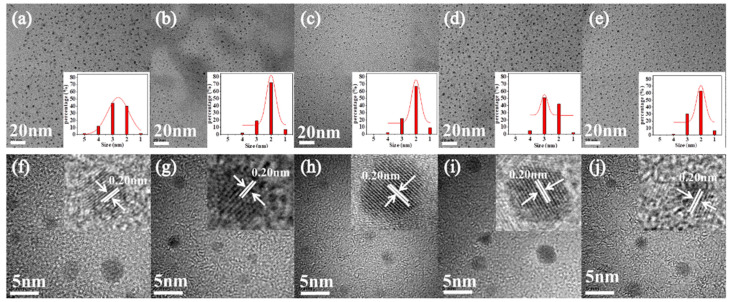
(**a**–**e**) TEM images of CDs (from left to right). The insets show histograms and Gaussian fits to the particle-size distributions of these CDs. (**f**–**j**) HR-TEM images of CDs (from left to right).

We examined the morphologies of the prepared samples using transmission electron microscopy (TEM). The TEM images of these CDs are shown in Figure 4a–e, respectively. Their morphologies are similar to those of spherical particles, and they showed good mono-dispersion. The average particle sizes of CDs-1 to CDs-5 were 3.0, 2.2, 2.3, 2.6, and 2.2 nm, respectively. Obviously, the similar particle size of the three samples suggests that the particle size was not the dominant mechanism responsible for the occurrence of chromatic emissions. Figure 4f–j exhibit high-resolution TEM (HR-TEM) images of CDs-1 to CDs-5, which display a well-resolved lattice spacing of 0.20 nm, which is close to the (100) diffraction from the crystal planes [34,35].

### 3.3. Solvent Tunable Emission

**Figure 5:**
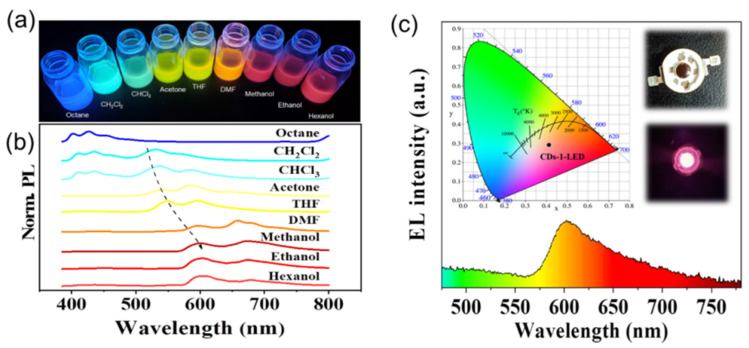
(**a**) Photographs of the same CDs-1 dispersed in nine different solvents and excited with λ = 365 nm UV irradiation. (**b**) The PL emission spectra of the same CDs-1. (**c**) The CIE 1931 containing the color coordinates of the CDs-1–LED device. Inset: The EL emission spectrum of the CDs-1–LED. The insets show optical images of the CDs-1–LED in the “off” (top) and “on” (bottom) states.

**Figure 6:**
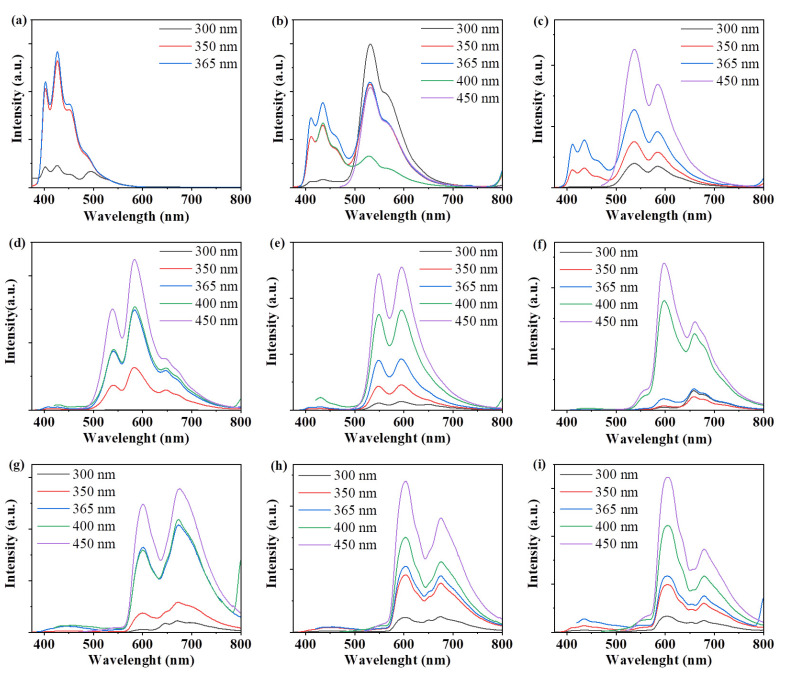
PL emission spectra of CDs-1 in (**a**) octane, (**b**) dichloromethane, (**c**) trichloromethane, (**d**) acetone, (**e**) tetrahydrofuran, (**f**) DMF, (**g**) methanol, (**h**) ethanol and (**i**) hexanol.

The CDs-1 was used as the research object to study the fluorescence phenomenon in different solvents. The CDs-1 have good solubility in octane, dichloromethane, chloroform, acetone, tetrahydrofuran, dimethylformamide, methanol, ethanol, hexanol, and other organic solvents. As shown in Figure 5a, the CDs-1 exhibit different fluorescence colors in different solvents at the same excitation wavelength (365 nm). The emission peaks were observed at 427 (22.2% of QY), 530 (10.7% of QY), 536 (4.2% of QY), 584 (26.4% of QY), 594 (9.8% of QY), 597 (31.6% of QY), 673 (28.1% of QY), 610 (26.4% of QY), and 679 (24.0% of QY) nm in octane, dichloromethane, trichloromethane, acetone, tetrahydrofuran, dimethylformamide, methanol, ethanol, and hexanol, respectively, corresponding to blue, cyan, yellow-green, orange, and red (Figure 5b). It can be seen from the previous two figures that the emission peak becomes red shifted with the increase in solvent polarity. This phenomenon is very similar to the “solvent discoloration” in organic dyes [36,37,38]. This solvation discoloration effect is usually attributed to intramolecular charge transfer [39,40]. Compared with existing reports of solvent-dependent CDs, the CDs-1 we prepared show the wider color-adjustable range of 427 nm–679 nm. In addition, it can be clearly seen from Figure 6 that when different excitation wavelengths are used, the fluorescence emission band of CDs-1 in any solvent will not move. This strict excitation wavelength independence is unique for CDs-1. For simplicity, the PL spectra of CDs-1 in dichloromethane and chloroform can be divided into two regions named the blue band (370 nm to 480 nm) and the yellow band (480 nm to 700 nm), respectively (Figure 6b,c). The blue band shows the classical properties related to excitation. According to previous reports, it can be divided into eigenstate emission and defect emission [41,42]. Under the high-energy excitation wavelength below 400 nm, there are two obvious bands of emission, and the final emission is cyan fluorescence, as shown in Figure 5a. Under the excitation wavelength above 400 nm, there is a single emission peak with 535 nm as the center, which is yellow fluorescence.

### 3.4. CDs-1 Was Applied to the Preparation of Red LED

From the unique optical properties of the CDs, the prepared CDs can be used as the luminescent materials in LEDs. We coated a CDs-1/epoxy composite onto a UV-LED chip that provided excitation light at 365 nm wavelength (see the experimental section for details). We dried the hybrid device in an 80 °C oven to produce a solid-state LED, as shown in the insets in Figure 5c, which shows the EL spectrum of a CDs-1–LED at 3.4 V. The maximum emission peak of the red LED is at 618 nm, and the red region accounts for 35.7% of the EL spectrum. Moreover, the CIE 1931 coordinates of the red LED device are located at (0.4175, 0.2936) (Figure 5c, inset). Note that the spectral center of the LED based on CDs-1 appears orange-red from the CIE 1931 coordinates.

### 3.5. The Fluorescence Quenching of CDs-1 by Fe^3+^

**Figure 7:**
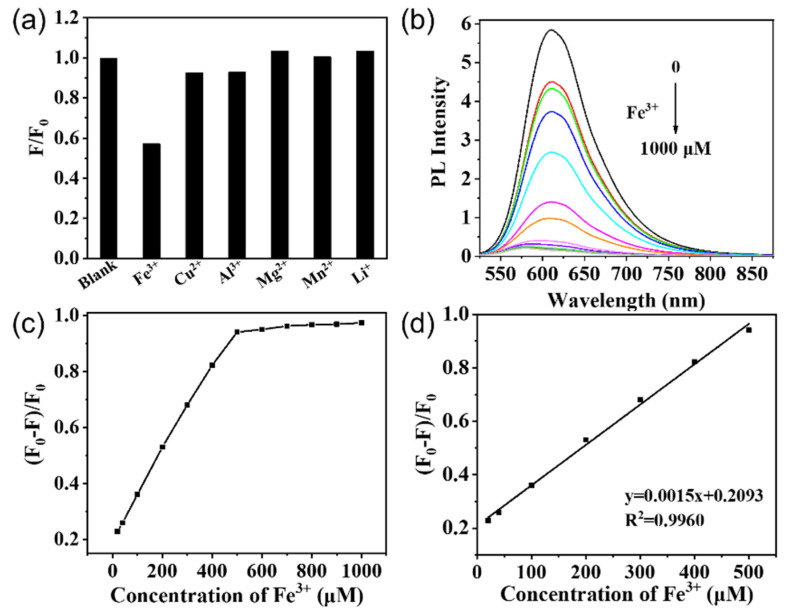
(**a**) Fluorescent responses of 100 μM CDs-1 to different metal ions. (**b**) The fluorescent response of CDs-1 to the addition of different concentrations of Fe^3+^. (**c**) Plots of the (F_0_ − F)/F_0_ with different concentrations of Fe^3+^. (**d**) The PL intensity F is linearly related to the concentration of Fe^3+^ over the ranges from 20–500 μM.

Detection of Fe^3+^ is necessary because these ions are highly toxic to organisms [43,44,45]; therefore, in this study we also determined the sensitivity of the CDs for detecting Fe^3+^. We used the prepared CDs-1 as a sensor to detect Fe^3+^ in aqueous solution. In addition, we investigated the selectivity of the CDs-1 to Fe^3+^ using Cu^2+^, Al^3+^, Mg^2+^, Mn^2+^, and Li^+^ as control experiments (Figure 7a). In the presence of Fe^3+^, the fluorescent intensity of the CDs was greatly reduced, whereas the effect of other ions on the fluorescent intensity was relatively weak. This shows that CDs have satisfactory selectivity for Fe^3+^ analysis. We believe that the fluorescence quenching mechanism of Fe^3+^ may be charge transfer and inhibited exciton recombination [46,47,48]. We believe that the interaction between the hydroxyl radicals on the surfaces of the CDs and Fe^3+^ changes the electronic structures of the CDs, resulting in the fluorescence quenching effect. This study determined the sensitivity of the CDs for detecting Fe^3+^. Figure 7b shows that the fluorescent intensity of the CDs decreases with increasing Fe^3+^ concentration, and in the presence of 500 μM Fe^3+^, the quenching efficiency can reach 94%.

Figure 7c,d present the relationship between the fluorescent quenching ratio (F_0_ − F)/F_0_ and the Fe^3+^ concentration, where F is the PL intensity, and F_0_ is the value for concentration zero. As the concentration of Fe^3+^ was varied from 20 μM to 500 μM, we found a good linear correlation, with the square of the correlation coefficient being R^2^ = 0.9960. We also obtained the linear regression equation y = 0.0015x + 0.2093. In addition, we found the limit of detection to be less than 20 μM, based on a signal-to-noise ratio of three from a simple iron-ion detection experiment.

## 4. Conclusions

In summary, we have reported the red luminescent CDs with high efficiency photoluminescence from 1, 4-diaminaphthalene. We used five different solvents as the reaction media for carbonization under the same conditions. The CDs prepared in octane and acetone had high fluorescence and QYs (26.4% and 17.9%, respectively). We also found a strong solvation effect of the CDs, and the CDs we prepared showed tunable fluorescent emission over the wide spectral range from 427 nm to 679 nm. Based on the quenching effect of Fe^3+^ on the PL emission from the CDs, we carried out a simple Fe^3+^ sensor detection application. In addition, based on its excitation behavior in solid polymer matrix, we prepared red LEDs coated with CDs/epoxy resin composites. This work provides a new method for developing red fluorescent CDs with tunable emission and high efficiency photoluminescence, and demonstrates great application potential in sensing and optoelectronic devices.

## Figures and Tables

**Table 1:** Elemental proportions and chemical bonds in different CDs.

	CDs-1	CDs-2	CDs-3	CDs-4	CDs-5
C1s	78.23%	82.27%	81.62%	77.44%	75.87%
O1s	13.28%	10.33%	9.88%	17.36%	13.20%
N1s	8.50%	7.40%	8.50%	5.21%	10.94%
C=C/C=N	88.00%	92.00%	83.00%	89.00%	81.00%
C–N/C–O	11.00%	6.00%	14.00%	10.00%	16.00%
C=O	1.00%	2.00%	3.00%	1.00%	3.00%

## Data Availability

The data presented in this study are available on request from the corresponding author.

## Sample Availability

Samples of the compounds are available from the authors.
